# Prediction of First-Year Corrosion Losses of Carbon Steel and Zinc in Continental Regions

**DOI:** 10.3390/ma10040422

**Published:** 2017-04-18

**Authors:** Yulia M. Panchenko, Andrey I. Marshakov

**Affiliations:** A.N. Frumkin Institute of Physical Chemistry and Electrochemistry, Russian Academy of Sciences, Moscow 119071, Russia; mar@ipc.rssi.ru

**Keywords:** carbon steel, zinc, modeling studies, atmospheric corrosion

## Abstract

Dose-response functions (DRFs) developed for the prediction of first-year corrosion losses of carbon steel and zinc (*K*_1_) in continental regions are presented. The dependences of mass losses on SO_2_ concentration, *K* = *f*([SO_2_]), obtained from experimental data, as well as nonlinear dependences of mass losses on meteorological parameters, were taken into account in the development of the DRFs. The development of the DRFs was based on the experimental data from one year of testing under a number of international programs: ISO CORRAG, MICAT, two UN/ECE programs, the Russian program in the Far-Eastern region, and data published in papers. The paper describes predictions of *K*_1_ values of these metals using four different models for continental test sites under UN/ECE, RF programs and within the MICAT project. The predictions of *K*_1_ are compared with experimental *K*_1_ values, and the models presented here are analyzed in terms of the coefficients used in the models.

## 1. Introduction

Predictions of the corrosion mass losses (*K*) of structural metals, in general for a period not exceeding 20 years, are made using the power function:

*K* = *K*_1_*τ*^n^, (1)

where *K*_1_ represents the corrosion losses for the first year, g/m^2^ or μm; *τ* is the test time in years; and *n* is a coefficient that characterizes the protective properties of corrosion products. The practical applications of Equation (1) for particular test locations in various regions of the world and the methods for *n* calculation are summarized in [1,2,3,4,5,6,7,8].

The power linear function that is believed to provide the most reliable predictions for any period of time and in any region of the world was suggested in [9,10]. Corrosion obeys a power law (Equation (1)) during an initial period and a linear law after the stationary stage starts. The total corrosion losses of metals for any period of time during the stationary stage can be calculated using Equation (2):

*K* = *K*_st_ + *α*(*τ* − *τ*_st_), (2)

where *K*_st_ stands for corrosion losses over the initial period calculated by Equation (1), g/m^2^ or μm; *τ*_st_ is the year when stabilization begins; and *α* is the yearly gain in corrosion losses of metals during the stationary stage in g/(m^2^year) or μm/year.

The differences in the predictions of corrosion losses by Equations (1) and (2) consist of different estimates of *τ*_st_, *α*, and *n* values for test locations with various corrosivity and atmosphere types. According to [10], *τ*_st_ equals 20 years. The *n* values are given per atmosphere type, irrespective of the atmosphere corrosivity within a particular type. In [9], *τ*_st_ = 6 years, and equations for *n* calculations based on the corrosivity of various atmosphere types are suggested. In [9,10], the *α* values are equal to the instantaneous corrosion rate at *τ*_st_.

Furthermore, various types of dose-response functions (DRFs) have been developed for long-term predictions of *K*; these can be used for certain territories or for any region of the world [11,12,13,14,15,16,17]. It should be noted that DRFs are power functions and have an advantage in that they provide predictions of first-year corrosion losses (*K*_1_) based on yearly-average meteorological and aerochemical atmosphere parameters. The power-linear function uses *K*_1_ values that should match the yearly-average corrosivity parameters of the test site atmosphere. The *K*_1_ values can be determined by repeated natural yearly tests in each location, which require significant expense and ISO 9223:2012(E) presents equations for the calculation of *K*_1_ of structural metals for any atmosphere types [18].

Recently, one-year and long-term predictions have been performed using models based on an artificial neural network (ANN) [19,20,21,22,23]. Their use is undoubtedly a promising approach in the prediction of atmospheric corrosion. The ANN “training” stage is programmed so as to obtain the smallest prediction error. Linear and nonlinear functions are used for *K* or *K*_1_ prediction by means of an ANN. Using an ANN, the plots of *K* (*K*_1_) versus specific corrosivity parameters can be presented visually as 2D or 3D graphs [19]. Despite the prospects of *K* prediction using ANNs, DRF development for certain countries (territories) is an ongoing task. The analytical form of DRFs is most convenient for application by a broad circle of experts who predict the corrosion resistance of materials in structures.

DRF development is based on statistical treatment, regression analysis of experimental data on *K*_1_, and corrosivity parameters of atmospheres in numerous test locations. All DRFs involve a prediction error that is characterized, e.g., by the *R*^2^ value or by graphical comparison in coordinates of predicted and experimental *K*_1_. However, comparisons of the results on *K*_1_ predictions based on different DRFs for large territories have not been available to date. Furthermore, the DRFs that have been developed assume various dependences of *K* on SO_2_ concentration; however, the shape of the *K* = *f*(SO_2_) function was not determined by analysis of data obtained in a broad range of atmosphere meteorological parameters.

The main purpose of this paper is to perform a mathematical estimate of the *K* = *f*(SO_2_) dependence for carbon steel and zinc, popular structural materials, and to develop new DRFs for *K*_1_ prediction based on the *K* = *f*(SO_2_) dependences obtained and the meteorological corrosivity parameters of the atmosphere. Furthermore, we will compare the *K*_1_ predictions obtained by the new and previously developed DRFs for any territories of the world, as well as analyze the DRFs based on the values of the coefficients in the equations.

## 2. Results

### 2.1. Development of DRFs for Continental Territories

To develop DRFs, we used the experimental data from all exposures for a one-year test period in continental locations under the ISO CORRAG international program [24], the MICAT project [11,25], the UN/ECE program [12,14], the Russian program [26], and the program used in [19]. The test locations for the UN/ECE program and the MICAT project are presented in Table 1. The corrosivity parameters of the test site atmospheres and the experimental *K*_1_ values obtained in four one-year exposures under the UN/ECE program are provided in Table 2, those obtained in three one-year exposures under the MICAT project are given in Table 3, and those obtained in the RF program are provided in Table 4. Cai et al. [19] report a selection of data from various literature sources. Of this selection, we use only the experimental data for continental territories that are shown in Table 5. The test results under the ISO CORRAG program [24] are not included in this paper because they lack the atmosphere corrosivity parameters required for *K*_1_ prediction. We used them simply to determine the *K* = *f*(SO_2_) dependences for steel and zinc.

### 2.2. Predictions of First-Year Corrosion Losses

To predict *K*_1_ for steel and zinc, we used the new DRFs presented in this paper (hereinafter referred to as “New DRFs”), in the standard [18] (hereinafter referred to as “Standard DRFs”), in [13] (hereinafter referred to as “Unified DRFs”), and the linear model [20] (hereinafter referred to as “Linear DRF”).

The Standard DRFs are intended for the prediction of *K*_1_ (*r*_corr_ in the original) in SO_2_- and Cl^−^-containing atmospheres in all climatic regions of the world. The *K*_1_ values are calculated in μm.

For carbon steel, Equation (3):

*K*_1_ = 1.77 × *P*_d_^0.52^ × exp(0.020 × *RH* + *f*_St_) + 0.102 × *S*_d_^0.62^ × exp(0.033 × *RH* + 0.040 × *T*), (3)

where *f*_St_ = 0.150·(*T* − 10) at *T* ≤ 10 °C; *f*_St_ = −0.054·(*T* − 10) at *T* > 10 °C.

For zinc, Equation (4):

*K*_1_ = 0.0129 × *P*_d_^0.44^ × exp(0.046 × *RH* + *f*_Zn_) + 0.0175 × *S*_d_^0.57^ × exp(0.008 × *RH* + 0.085 × *T*), (4)

where *f*_Zn_ = 0.038 × (*T* − 10) at *T* ≤ 10 °C; *f*_Zn_ = −0.071 × (*T* − 10) at *T* > 10 °C, where *T* is the temperature (°C) and *RH* (%) is the relative humidity of air; *P*_d_ and *S*_d_ are SO_2_ and Cl^−^ deposition rates expressed in mg/(m^2^day), respectively.

In Equations (3) and (4), the contributions to corrosion due to SO_2_ and Cl^−^ are presented as separate components; therefore, only their first components were used for continental territories.

Unified DRFs are intended for long-term prediction of mass losses *K* (designated as *ML* in the original) in SO_2_-containing atmospheres in all climatic regions of the Earth. It is stated that the calculation is given in g/m^2^.

For carbon steel, Equation (5):

*K* = 3.54 × [SO_2_]^0.13^ × exp{0.020 × *RH* + 0.059 × (*T*-10)} × *τ*^0.33^*T* ≤ 10 °C; *K* = 3.54 × [SO_2_]^0.13^ × exp{0.020 × *RH* − 0.036 × (*T*-10)} × *τ*^0.33^*T* > 10 °C. (5)

For zinc, Equation (6):

*K* = 1.35 × [SO_2_]^0.22^ × exp{0.018 × *RH* + 0.062 × (*T*-10)} × *τ*^0.85^ + 0.029 × Rain[H^+^] × *τ T* ≤ 10 °C; *K* = 1.35 × [SO_2_]^0.22^ × exp{0.018 × *RH* − 0.021 × (*T*-10)} × *τ*^0.85^ + 0.029 × Rain[H^+^] × *τ T* > 10 °C. (6)

where *T* is the temperature (°C) and *RH* (%) is the relative humidity of air; [SO_2_] is the concentration of SO_2_ in μg/m^3^; “Rain” is the rainfall amount in mm/year; [H^+^] is the acidity of the precipitation; and *τ* is the exposure time in years.

To predict the first-year corrosion losses, *τ* = 1 was assumed.

The standard DRFs and Unified DRFs were developed on the basis of the results obtained in the UN/ECE program and MICAT project using the same atmosphere corrosivity parameters (except from Rain[H^+^]). If *τ* = 1, the models have the same mathematical form and only differ in the coefficients. Both models are intended for *K*_1_ predictions in any regions of the world, hence it is particularly interesting to compare the results of *K*_1_ predictions with actual data.

The linear model was developed for SO_2_- and Cl^−^-containing atmospheres. It is based on the experimental data from the MICAT project only and relies on an artificial neural network. It is of special interest since it has quite a different mathematical form and uses different parameters. In the MICAT project, the air temperature at the test sites is mainly above 10 °C (Table 3). Nevertheless, we used this model, like the other DRFs, also for test locations with any temperatures.

The first-year corrosion losses of carbon steel (designated as “Fe” in the original) are expressed as Equation (7):

*K*_1_ = b_0_ + Cl^−^ × (b_1_ + b_2_ × P + b_3_ × *RH*) + b_4_ × *TOW* × [SO_2_], (7)

where b_0_ = 6.8124, b_1_ = −1.6907, b_2_ = 0.0004, b_3_ = 0.0242, and b_4_ = 2.2817; *K*_1_ is the first-year corrosion loss in μm; Cl^−^ is the chloride deposition rate in mg/(m^2^·day); P is the amount of precipitation in mm/year; *RH* is the air relative humidity in %; *TOW* is the wetting duration expressed as the fraction of a year; and [SO_2_] is the SO_2_ concentration in μg/m^3^. The prediction results for the first year are expressed in μm.

To predict *K*_1_ in continental regions, only the component responsible for the contribution to corrosion due to SO_2_ was used.

The *K*_1_ values in μm were converted to g/m^2^ using the specific densities of steel and zinc, 7.8 and 7.2 g/cm^3^, respectively. Furthermore, the relationship *P*_d,p_ mg/(m^2^·day) = 0.67 *P*_d,c_ μg/m^3^ was used, where *P*_d,p_ is the SO_2_ deposition rate and *P*_d,c_ is the SO_2_ concentration [18].

The calculation of *K*_1_ is given for continental test locations at background Cl^−^ deposition rates ≤2 mg/(m^2^·day) under UN/ECE and RF programs and MICAT project. The R^2^ values characterizing the prediction results as a whole for numerous test locations are not reported here. The *K*_1_ predictions obtained were compared to the experimental values of *K*_1_ for each test location, which provides a clear idea about the specific features of the DRFs.

## 3. Results

### 3.1. DRF Development

Corrosion of metals in continental regions depends considerably on the content of sulfur dioxide in the air. Therefore, development of a DRF primarily requires that this dependence, i.e., the mathematical relationship *K* = *f*(SO_2_), be found. The dependences reported in graphical form in [20,27] differ from each other. The relationship is non-linear, therefore the decision should be made on which background SO_2_ concentration should be selected, since the calculated *K*_1_ values would be smaller than the experimental ones at [SO_2_] <1 if non-linear functions are used. [SO_2_] values <1 can only be used in linear functions. The background values in Table 2, Table 3 and Table 4 are presented as “Ins.” (Insignificant), ≤1, 3, 5 μg/m^3^, which indicates that there is no common technique in the determination of background concentrations. For SO_2_ concentrations of “Ins.” or ≤1 μg/m^3^, we used the value of 1 μg/m^3^, whereas the remaining SO_2_ concentrations were taken from the tables.

In finding the *K* = *f*(SO_2_) relationship, we used the actual test results of all first-year exposures under each program rather than the mean values, because non-linear functions are also used.

**Figure 1:**
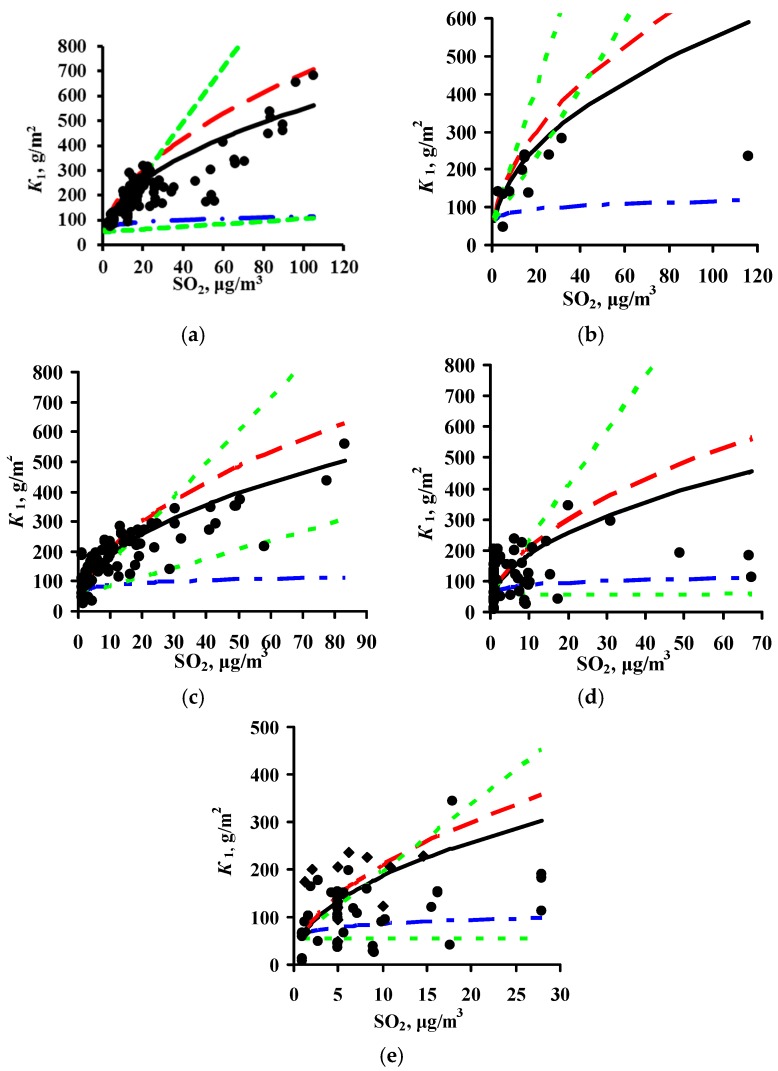
Dependence of first-year corrosion losses of steel (*K*_1_) on SO_2_ concentration based on data from ISO CORRAG program (**a**), Ref. [19] (**b**), UN/ECE program (**c**), MICAT project (**d**), and data from MICAT project cited in [20] (**e**). ▬▬—*α* = 0.47 (New DRF), ▬ ▬—*α* = 0.52 (Standard DRF), ▬●▬—*α* = 0.13 (Unified DRF), ─ ─ ─—model [20] for TOW ranges in accordance with the data in Table 2, Table 3, Table 4 and Table 5.

**Figure 2:**
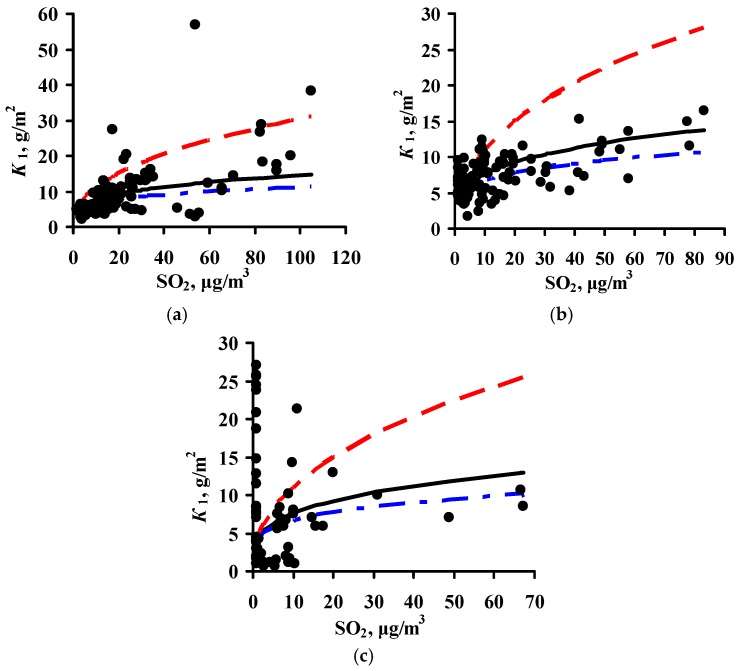
Dependence of first-year corrosion losses of zinc (*K*_1_) on SO_2_ concentration based on the data from ISO CORRAG program (**a**), UN/ECE program (**b**), and MICAT project (**c**). ▬▬—*α* = 0.28 (New DRF), ▬ ▬—*α* = 0.44 (Standard DRF), ▬●▬—*α* = 0.22 (Unified DRF).

The *K* = *f*(SO_2_) relationships obtained for each program are shown in Figure 1 for steel and in Figure 2 for zinc. In a first approximation, this relationship can be described by the following function for experimental *K*_1_ values obtained in a broad range of meteorological atmosphere parameters:

*K*_1_ = *K*_1_° × [SO_2_]*^α^*, (8)

where *K*_1_° are the average corrosion losses over the first year (g/m^2^) in a clean atmosphere for the entire range of *T* and *RH* values; and *α* is the exponent that depends on the metal.

The *K*_1_° values corresponding to the mean values of the parameter range of climatic conditions in clean atmospheres were found to be the same for the experimental data of all programs, namely, 63 and 4 g/m^2^, while *α* = 0.47 and 0.28 for carbon steel and zinc, respectively. A similar *K*_1_° value for carbon steel was also obtained from the Linear DRF, Equation (6). In fact, at background SO_2_ concentrations = 1 μg/m^3^ in PE4 test location (Table 3) at TOW = 26 h/year (0.002 of the year), the calculated *K*_1_° is to 53 g/m^2^, while for CO_2_ test location at TOW = 8760 h/year (entire year) it is 71 g/m^2^; the mean value is 62 g/m^2^.

Based on Equation (8), it may be accepted in a first approximation that the effect of [SO_2_] on corrosion is the same under any climatic conditions and this can be expressed in a DRF by an [SO_2_]*^α^* multiplier, where *α* = 0.47 or *α* = 0.28 for steel or zinc, respectively. The *K*_1_° values in Equation (8) depend on the climatic conditions and are determined for each test location based on the atmosphere meteorological parameters.

In the development of New DRF, the *K*_1_ values were determined using the DRF mathematical formula presented in the Standard DRF and in the Unified DRF, as well as meteorological parameters *T*, *RH*, and *Prec* (*Rain* for warm climate locations or *Prec* for cold climate locations). The complex effect of *T* was taken into account: corrosion losses increase with an increase in *T* to a certain limit, *T*_lim_; its further increase slows down the corrosion due to radiation heating of the surface of the material and accelerated evaporation of the adsorbed moisture film [12,28]. It has been shown [29] that *T*_lim_ is within the range of 9–11 °C. Similarly to Equations (3)–(6), it is accepted that *T*_lim_ equals 10 °C. The need to introduce *Prec* is due to the fact that in northern RF regions, the *K*_1_ values are low at high *RH*, apparently owing not only to low *T* values but also to the small amount of precipitation, including solid precipitations. The values of the coefficients reflecting the effect of *T*, *RH* and *Prec* on corrosion were determined by regression analysis.

The New DRFs developed for the prediction of *K*_1_ (g/m^2^) for the two temperature ranges have the following forms:

for carbon steel :

*K*_1_ = 7.7 × [SO_2_]^0.47^ × exp{0.024 × *RH* + 0.095 × (*T*-10) + 0.00056 × *Prec*} *T* ≤ 10 °C; *K*_1_ = 7.7 × [SO_2_]^0.47^ × exp{0.024 × *RH* − 0.095 × (*T*-10) + 0.00056 × *Prec*} *T* > 10 °C, (9)

and for zinc:

*K*_1_ = 0.71 × [SO_2_]^0.28^ × exp{0.022 × *RH* + 0.045 × (*T*-10) + 0.0001 × *Prec*} *T* ≤ 10 °C; *K*_1_ = 0.71 × [SO_2_]^0.28^ × exp{0.022 × *RH* − 0.085 × (*T*-10) + 0.0001 × *Prec*} *T* > 10 °C. (10)

### 3.2. Predictions of K_1_ Using Various DRFs for Carbon Steel

Predictions of *K*_1_ were performed for all continental test locations with chloride deposition rates ≤2 mg/(m^2^·day). The results of *K*_1_ prediction (*K*_1_^pr^) from Equations (3)–(7), (9), and (10) are presented separately for each test program. To build the plots, the test locations were arranged by increasing experimental *K*_1_ values (*K*_1_^exp^). Their sequence numbers are given in Table 2, Table 3 and Table 4. The increase in *K*_1_ is caused by an increase in atmosphere corrosivity due to meteorological parameters and SO_2_ concentration. All the plots are drawn on the same scale. All plots show the lines of prediction errors *δ* = ±30% (the 1.3 *K*_1_^exp^–0.7 *K*_1_^exp^ range). This provides a visual idea of the comparability of *K*_1_^pr^ with *K*_1_^exp^ for each DRF. The scope of this paper does not include an estimation of the discrepancy between the *K*_1_^pr^ values obtained using various DRFs with the *K*_1_^exp^ values obtained for each test location under the UN/ECE and RF programs. The scatter of points is inevitable. It results from the imperfection of each DRF and the inaccuracy of experimental data on meteorological parameters, SO_2_ content, and *K*_1_^exp^ values. Let us just note the general regularities of the results on *K*_1_^pr^ for each DRF.

**Figure 3:**
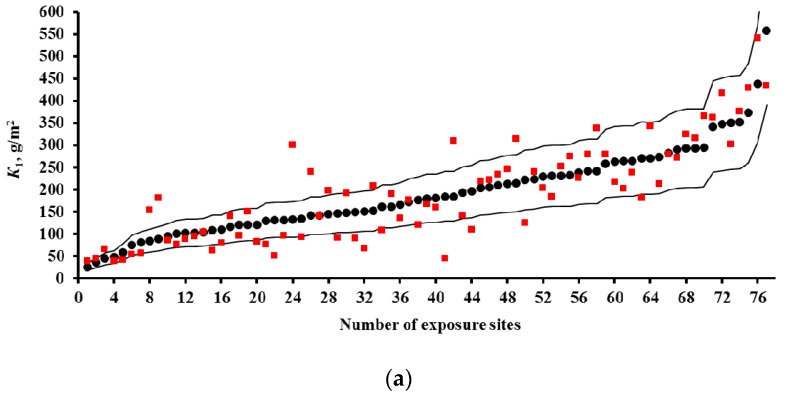

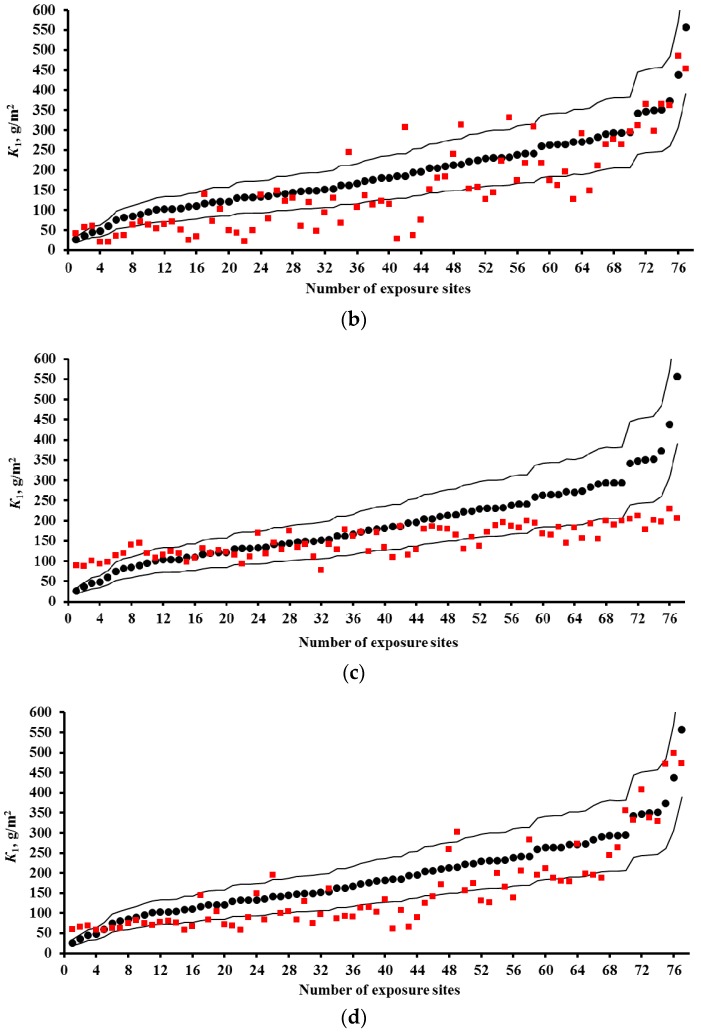
Carbon steel. UN/ECE program. *K*_1_ predictions by the New DRF (**a**); Standard DRF (**b**); Unified DRF (**c**); and Linear DRF [20] (**d**). ●—experimental *K*_1_ data; ■—*K*_1_ predictions. Thin lines show the calculation error (± 30%). The numbers of the exposure sites are given in accordance with Table 2.

**Figure 4:**
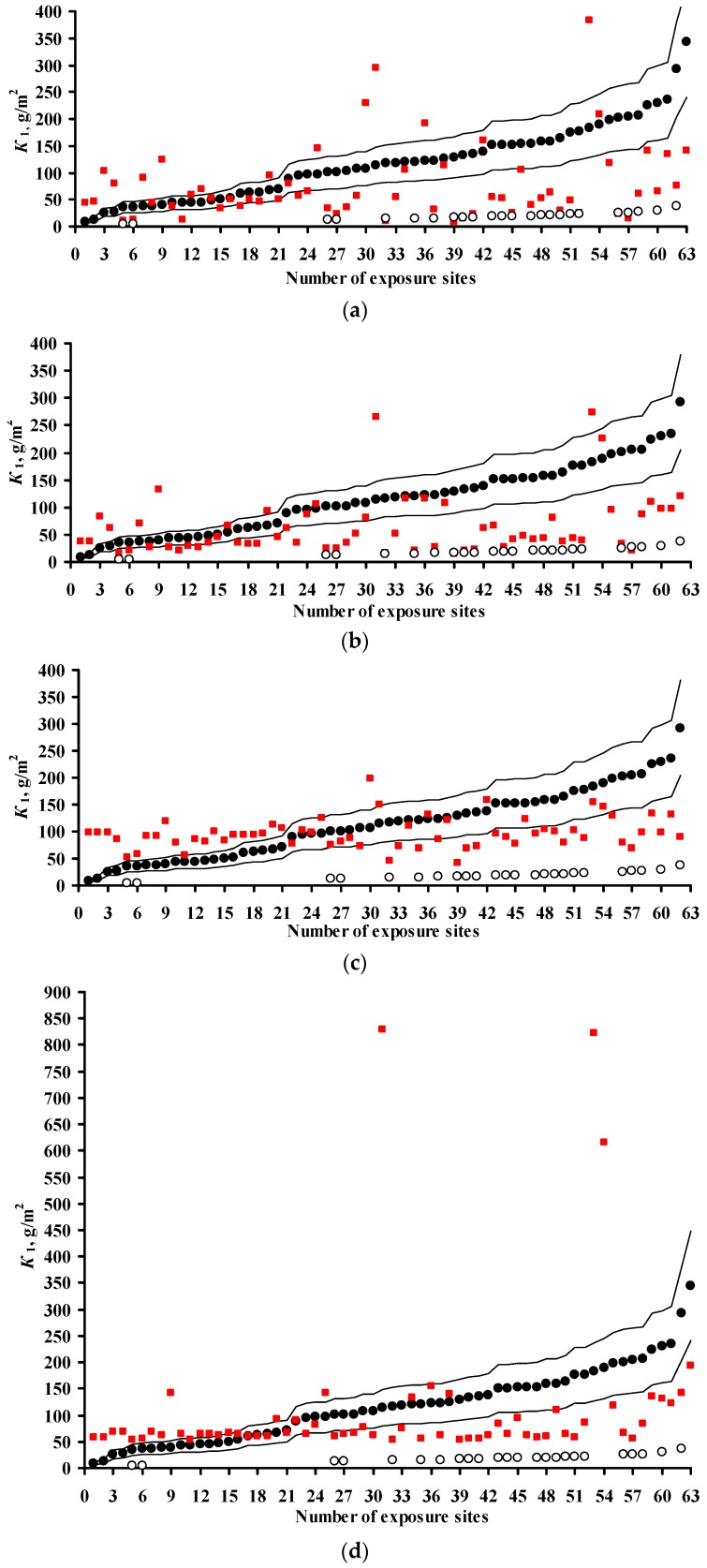

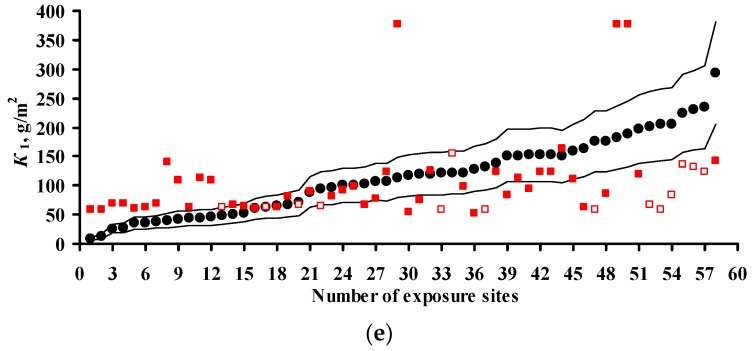
Carbon steel. MICAT program. *K*_1_ predictions by the New DRF (**a**); Standard DRF (**b**); Unified DRF (**c**); linear model [20] (**d**); and linear model based on data from [20] (**e**). ●—experimental *K*_1_ data; ■—*K*_1_ predictions; □—the test locations in [20] which were not used (only for Figure 4e); ○—experimental *K*_1_ data under the assumption that they were expressed in g/m^2^ rather than in μm. Thin lines show the calculation error (±30%). The numbers of the exposure sites are given in accordance with Table 3.

**Figure 5:**
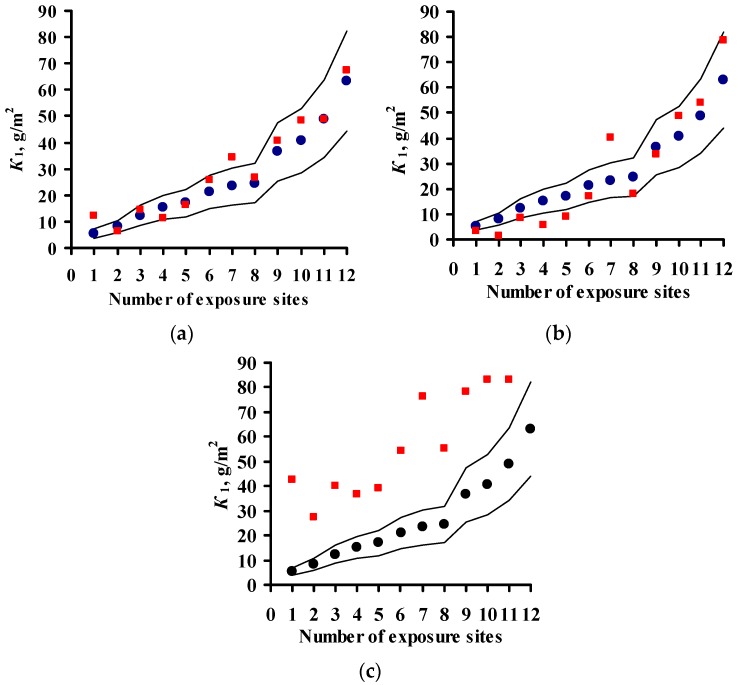
Carbon steel. RF program. *K*_1_ predictions by the New DRF (**a**); Standard DRF (**b**); and Unified DRF (**c**). ●—experimental *K*_1_ data; ■—*K*_1_ predictions. Thin lines show the calculation error (±30%). The numbers of the exposure sites are given in accordance with Table 4.

The results on *K*_1_^pr^ for carbon steel for the UN/ECE program, MICAT project, and RF program are presented in Figure 3, Figure 4 and Figure 5, respectively. It should be noted that according to the Unified DRF (Equation (5)), the *K*_1_^pr^ of carbon steel in RF territory [30] had low values. It was also found that the *K*_1_^pr^ values are very low for the programs mentioned above. Apparently, the *K*_1_^pr^ values (Equation (5)) were calculated in μm rather than in g/m^2^, as the authors assumed. To convert *K*_1_^pr^ in μm to *K*_1_^pr^ in g/m^2^, the 3.54 coefficient in Equation (6) was increased 7.8-fold.

In the UN/ECE program, the *K*_1_^pr^ values match *K*_1_^exp^ to various degrees; some *K*_1_^pr^ values exceed the error *δ* (Figure 3). Let us describe in general the locations in which *K*_1_^pr^ values exceed *δ*. For the New DRFs (Figure 3a) there are a number of locations with overestimated *K*_1_^pr^ and with underestimated *K*_1_^pr^ values at different atmosphere corrosivities. For the Standard DRF (Figure 3b) and Linear DRF (Figure 3d), locations with underestimated *K*_1_^pr^ values prevail, also at different *K*_1_^exp^. For the Unified DRF (Figure 3c), *K*_1_^pr^ are overestimated for locations with small *K*_1_^exp^ and underestimated for locations with high *K*_1_^exp^. The possible reasons for such regular differences for *K*_1_^pr^ from *K*_1_^exp^ will be given based on an analysis of the coefficients in the DRFs.

For the MICAT project, *K*_1_^pr^ considerably exceeds *δ* for all DRFs in many locations (Figure 4). Overestimated and considerably overestimated *K*_1_^pr^ values are mainly observed in locations with small *K*_1_^exp^, while underestimated *K*_1_^pr^ values are mainly observed for locations with high *K*_1_^exp^. Furthermore, for the Linear DRF (Figure 4d), particularly overestimated values are observed in location B6 (No. 31, No. 53, and No. 54) at all exposures. This test location should be noted. The corrosivity parameters under this program reported in [20] are different for some test locations (Table 3). In fact, for B6, the [SO_2_] value for all exposures is reported to be 28 μg/m^3^ instead of 67.2; 66.8 and 48.8 μg/m^3^. Figure 4e presents *K*_1_^pr^ for the Linear DRF with consideration for the parameter values reported in [20]. Naturally, *K*_1_^pr^ for B6 decreased considerably in comparison with the values in Figure 4d but remained rather overestimated with respect to *K*_1_^exp^.

If all DRFs give underestimated *K*_1_^pr^ values for the same locations, this may result from an inaccuracy of experimental data, i.e., corrosivity parameters and/or *K*_1_^exp^ values. We did not perform any preliminary screening of the test locations. Therefore, it is reasonable to estimate the reliability of *K*_1_^exp^ only in certain locations by comparing them with other locations. Starting from No. 26, *K*_1_^pr^ values are mostly either smaller or considerably smaller than *K*_1_^exp^. The locations with underestimated *K*_1_^pr^ that are common to all DRFs include: A4 (No. 5, No. 6), B1 (No. 28), B10 (No. 26), B11 (No. 41), E1 (No. 47, No. 48, No. 51), E4 (No. 43, 49, 50), EC1 (No. 45, No. 52, No. 56), CO3 (No. 40, 57), PE4 (No. 32, No. 39), M3 (No. 58, No. 60, No. 62). To perform the analysis, Table 6 was composed. It contains the test locations that, according to our estimates, have either questionable or reliable *K*_1_^exp^ values. It clearly demonstrates the unreliability of *K*_1_^exp^ in some test locations. For example, in the test locations PE4 and A4, with *RH* = 33%–51% and *TOW* = 0.003–0.114 of the year at background [SO_2_], *K*_1_^exp^ are 4.5–16.5 μm (35.1–117 g/m^2^), while under more corrosive conditions in E8 and M2 with *RH* = 52%–56% and *TOW* = 0.100–0.200 of the year and [SO_2_] = 6.7–9.9 μg/m^3^, *K*_1_^exp^ values are also 3.3–15.2 μm (25.7–118.6 g/m^2^). The impossibility of high *K*_1_ values in PE4 and A4 is also confirmed by the 3D graph of the dependence of *K* on SO_2_ and *TOW* in [20]. Alternatively, for example, in B1, CO3 and B11 with *RH* = 75%–77% and *TOW* = 0.172–0.484 of the year and [SO_2_] = 1–1.7 μg/m^3^, *K*_1_^exp^ = 13.1–26.2 μm (102.2–204.4 g/m^2^), whereas in A2 and A3 with *RH* = 72%–76% and *TOW* = 0.482–0.665 of the year and [SO_2_] = 1–10 μg/m^3^, *K*_1_^exp^ is as small as 5.6–16.1 μm (43.7–125.6 g/m^2^). The *K*_1_ values reported for locations with uncertain data are 2–4 times higher than the *K*_1_ values in trusted locations. The reason for potentially overestimated *K*_1_^exp^ values being obtained is unknown. It may be due to non-standard sample treatment or to corrosion-related erosion. It can also be assumed that the researchers (performers) reported *K*_1_ in g/m^2^ rather than in μm. If this assumption is correct, then *K*_1_^pr^ values would better match *K*_1_^exp^ (Figure 4). Unfortunately, we cannot compare the questionable *K*_1_^exp^ values with the *K*_1_^exp^ values rejected in the study where an artificial neural network was used [20]. We believe that, of the *K*_1_^exp^ values listed, only the data for the test locations up to No. 26 in Figure 4 can be deemed reliable.

For the RF program, the *K*_1_^pr^ values determined by the New DRF and the Standard DRF are pretty comparable with *K*_1_^exp^, but they are considerably higher for the Unified DRF (Figure 5).

The presented figures indicate that all DRFs which have the same parameters but different coefficients predict *K*_1_ for same test locations with different degrees of reliability. That is, combinations of various coefficients in DRFs make it possible to obtain *K*_1_^pr^ results presented in Figure 3, Figure 4 and Figure 5. In view of this, the analysis of DRFs in order to explain the principal differences of *K*_1_^pr^ from *K*_1_^exp^ for each DRF appears interesting.

### 3.3. Analysis of DRFs for Carbon Steel

The DRFs were analyzed by comparison of the coefficients in Equations (3), (5) and (9). Nonlinear DRFs can be represented in the form:

*K*_1_ = *A* × [SO_2_]*^α^*exp{*k*_1_ × *RH* + *k*_2_ × (*T*−10) + *k*_3_ × *Prec*}

or

*K*_1_ = *A* × [SO_2_]*^α^* × e^*k*1·*RH*^ × e^*k*2·(*T*−10)^ × e^*k*3·*Prec*^,

where *A* × e*^k^*^1·*RH*^ × e*^k^*^2·(*T*−10)^ × e*^k^*^3·*Prec*^ = *K*_10_.

The values of the coefficients used in Equations (3), (5) and (9) are presented in Table 7.

To compare the *α* values, *K*_1_° = 63 g/m^2^ at [SO_2_] = 1 μg/m^3^ was used in Equation (8) for all DRFs. The [SO_2_]*^α^* plots for all the DRFs for all programs are presented in Figure 1. For the New DRF, the line *K* = *f*(SO_2_) was drawn approximately through the mean experimental points from all the test programs. Therefore, one should expect a uniform distribution of error *δ*, e.g., in Figure 3a. For the Standard DRF, *α* = 0.52 is somewhat overestimated, which may result in more overestimated *K*_1_ values at high [SO_2_]. However, in Figure 3b for CS1 (No. 76), CS3 (No. 73, 74, 77) and GER10 (No. 76), *K*_1_^pr^ overestimation is not observed, apparently due to effects from other coefficients in DRF. For Unified DRF *α* = 0.13, which corresponds to a small range of changes in *K*_1_ as a function of SO_2_. Therefore, in Figure 3c and Figure 4c, the *K*_1_^pr^ present a nearly horizontal band that is raised to the middle of the *K*_1_^exp^ range due to a higher value of *A* = 3.54 μm (27.6 g/m^2^), Table 7. As a result, the Unified DRF cannot give low *K*_1_^pr^ values for rural atmospheres, Figure 3c and Figure 5c, or high *K*_1_^pr^ values for industrial atmospheres, Figure 3c.

For the Linear DRF we present *K*_1_^pr^—[SO_2_] plots for *TOW* (fraction of a year) within the observed values: 0.043–0.876 for ISO CORRAG program; 0.5–1 based on the data in [19]; 0.17–0.62 from UN/ECE program; 0.003–1 from the MICAT project, and 0.002–0.8 based on the data [20] for the MICAT project, Figure 1. One can see that reliable *K*_1_^pr^ are possible in a limited range of *TOW* and [SO_2_]. The *K*_1_^pr^ values are strongly overestimated at high values of these parameters (Figure 4c,d). That is, the Linear model has a limited applicability at combinations of *TOW* and [SO_2_] that occur under natural conditions. Furthermore, according to the Linear DRF, the range of *K*_1_^pr^ in clean atmosphere is 53–71 g/m^2^, therefore the *K*_1_^pr^ values in clean atmosphere lower than 53 g/m^2^ (Figure 3d and Figure 4d,e) or above 71 g/m^2^ cannot be obtained. Higher *K*_1_^pr^ values can only be obtained due to [SO_2_] contribution. The underestimated *K*_1_^pr^ values in comparison with *K*_1_^exp^ for the majority of test locations (Figure 3d) are apparently caused by the fact that the effects of other parameters, e.g., *T*, on corrosion are not taken into account.

**Figure 6:**
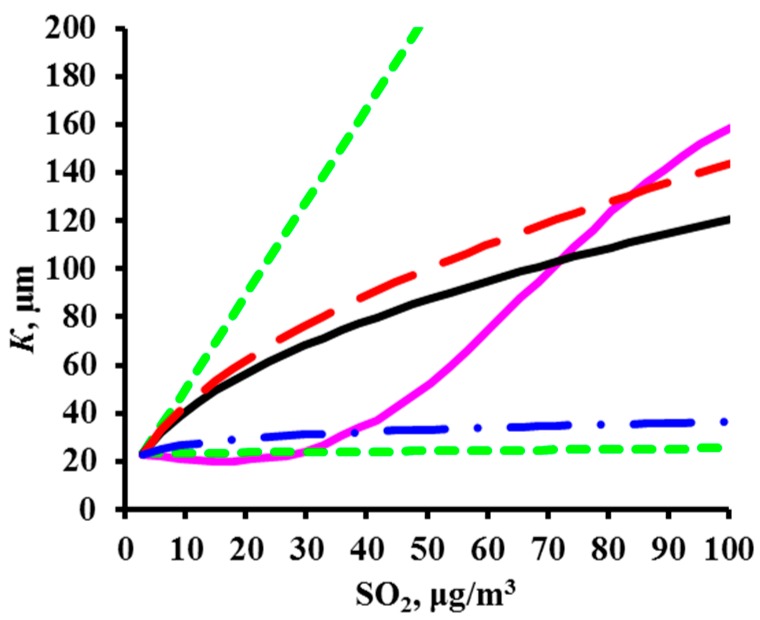
Comparison of *K* = *f*(SO_2_) plots for the DRF presented in [20]. ▬ ▬ plot according to [20], ▬▬ by the New DRF; ▬ ▬ by the Standard DRF; ▬•▬ by the Unified DRF; - - - by the Linear DRF [20].

Figure 6 compares *K* = *f*(SO_2_) for all the models with the graphical representation of the dependence reported in [20] (for [SO_2_], mg/(m^2^·d) values were converted to μg/m^3^). The dependence in [20] is presented for a constant temperature, whereas the dependences given by DRFs are given for average values in the entire range of meteorological parameters in the test locations. Nevertheless, the comparison is of interest. Below 70 and 80 μg/m^3^, according to [20], *K* has lower values than those determined by the New DRF and Standard DRF, respectively, while above these values, *K* has higher values. According to the Unified DRF, *K* has extremely low values at all [SO_2_] values, whereas according to the Linear DRF (TOW from 0.03 to 1), the values at *TOW* = 1 are extremely high even at small [SO_2_].

**Figure 7:**
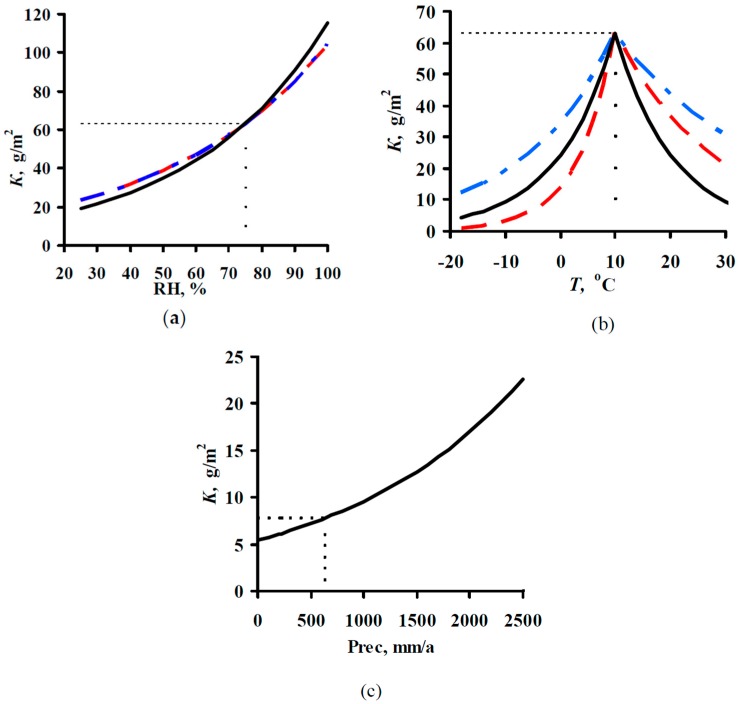
Variation of *K* for carbon steel vs. relative humidity (**a**), temperature (**b**). and *Prec* (**c**) with account for the values of the DRF coefficients. ▬▬ by the New DRF; ▬ ▬ by the Standard DRF; ▬•▬ by the Unified DRF.

To perform a comparative estimate of *k*_1_ and *k*_2_, let us use the value *T*_lim_ = 10 °C accepted in the DRF, i.e., where the temperature dependence changes. Furthermore, it is necessary to know the *K*_1_ value in clean atmosphere at *T*_lim_ and at the *RH* that is most common at this temperature. These data are unknown at the moment. Therefore, we’ll assume that at *T*_lim_ = 10 °C and *RH* = 75%, *K* = 63 g/m^2^. The dependences of *K* on *T* and *RH* under these conditions and with consideration for the corresponding *k*_1_ and *k*_2_ for each DRF are presented in Figure 7.

The nearly coinciding *k*_1_ values (0.020 for the Unified DRF and Standard DRF, and 0.024 for the New DRF, Table 8) result in an insignificant difference in the *RH* effect on *K* (Figure 7a).

The temperature coefficient *k*_2_ has a considerable effect on *K*. For the Unified *DRF*, the *k*_2_ values of 0.059 (−0.036) for *T* ≤ 10 °C (*T* > 10 °C) create the lowest decrease in *K* with a *T* decrease (increase) in comparison with the other DRFs (Figure 7b). A consequence of such *k*_2_ values can be demonstrated by examples. Due to the temperature effect alone, *K* ~ 15 g/m^2^ at *T* = −12 °C (Figure 7b) and *K*~45 g/m^2^ at *T* = 20 °C. The effects of other parameters and account for the *A* value would result in even more strongly overestimated *K*^pr^ values. For comparison: in Bilibino at *T* = −12.2 °C and *RH* = 80%, *K*_1_^exp^ = 5.4 g/m^2^ (Table 4) and *K*^pr^ = 42 g/m^2^ (Figure 5). In A3 test location, at *T* = 20.6 °C and *RH* = 76%, *K*_1_^exp^ = 44.5 g/m^2^ (Table 4), while due to *A* and other parameters, *K*_1_^pr^ = 86.2 g/m^2^, Figure 4c.

In the Standard DRF, the *k*_2_ values are higher than in the Unified DRF: 0.150 and −0.054 for *T* ≤ 10 °C and *T* > 10 °C, respectively, so a greater *K* decrease is observed, especially at *T* ≤ 10 °C, Figure 7b. At low *T*, the *K* values are small, e.g., *K* ~ 2 g/m^2^ at *T* = −12 °C. In *K*_1_^pr^ calculations, the small *K* are made higher due to *A*, and they are higher in polluted atmospheres due to higher *α* = 0.52. As a result, *K*^pr^ are quite comparable with *K*^exp^, Figure 3b. However, let us note that *K*^pr^ is considerably lower than *K*^exp^ in many places. Perhaps, this is due to an abrupt decrease in *K* in the range *T* ≤ 10 °C. This temperature range is mostly met in test locations under the UN/ECE program.

In the New DRF, *k*_2_ has an intermediate value at *T* ≤ 10 °C and the lowest value at *T* > 10 °C, whereas *A* has the lowest value. It is more difficult to estimate the *k*_2_ value with similar *k*_2_ values in the other DRFs, since the New DRF uses one more member, i.e., e*^k^*^3·*Prec*^. The dependence of *K* on *Prec* is presented in Figure 7c. The following arbitrary values were used to demonstrate the possible effect of *Prec* on *K*: *K* = 7.8 g/m^2^ at *Prec* = 632 mm/year. For example, in location PE5 (UN/ECE program) with *Prec* = 632 mm/year, *K* = 7.8 g/m^2^ at *T* = 12.2 °C and *RH* = 67%. The maximum *Prec* was taken as 2500 mm/year, e.g., it is 2144 mm/year in NOR23 (UN/ECE program) and 2395 mm/year in B8 (MICAT project). It follows from the figure that, other conditions being equal, *K* can increase from 5.4 to 22.6 g/m^2^ just due to an increase in *Prec* from 0 to 2500 mm/year at *k*_3_ = 0.00056 (Table 7).

Thus, it has been shown that the coefficients for each parameter used in the DRFs vary in rather a wide range. The most reliable *K*_1_^pr^ can be reached if, in order to find the most suitable coefficients, the DRFs are based on the *K* = *f*(SO_2_) relationship obtained.

### 3.4. Predictions of K_1_ Using Various DRFs for Zinc

**Figure 8:**
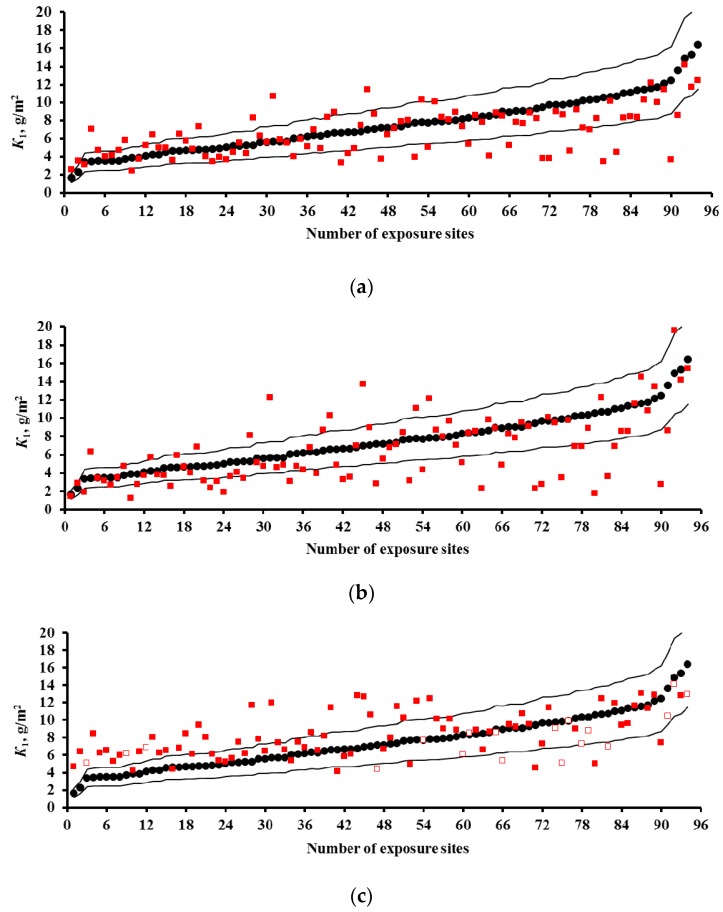
Zinc. UN/ECE program. *K*_1_ predictions by the New DRF (**a**); Standard DRF (**b**); and Unified DRF (**c**). ●—experimental *K*_1_ data; ■—*K*_1_ predictions. □—*K*_1_ predictions without taking [H^+^] into account (only for the Unified DRF). Thin lines show the calculation error (±30%). The numbers of the exposure sites are given in accordance with Table 2.

**Figure 9:**
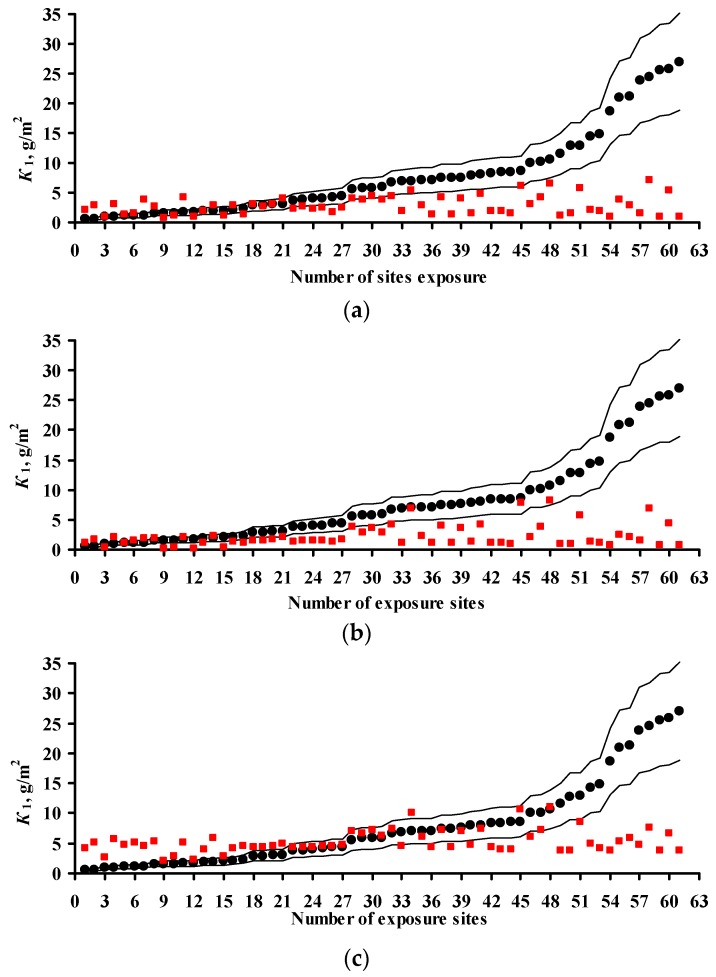
Zinc. MICAT program. *K*_1_ predictions by the New DRF (**a**); Standard DRF (**b**); and Unified DRF (**c**). ●—experimental *K*_1_ data; ■—*K*_1_ predictions. Thin lines show the calculation error (± 30%). The numbers of the exposure sites are given in accordance with Table 3.

**Figure 10:**
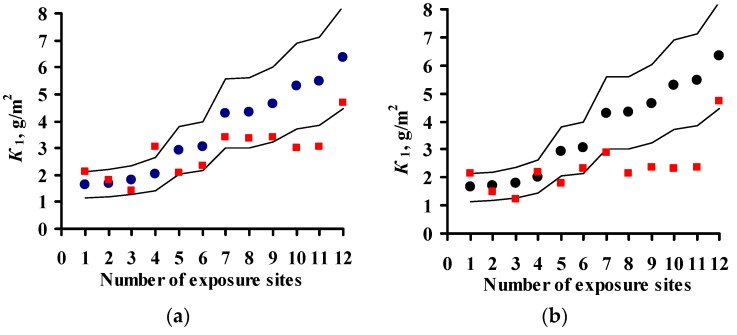

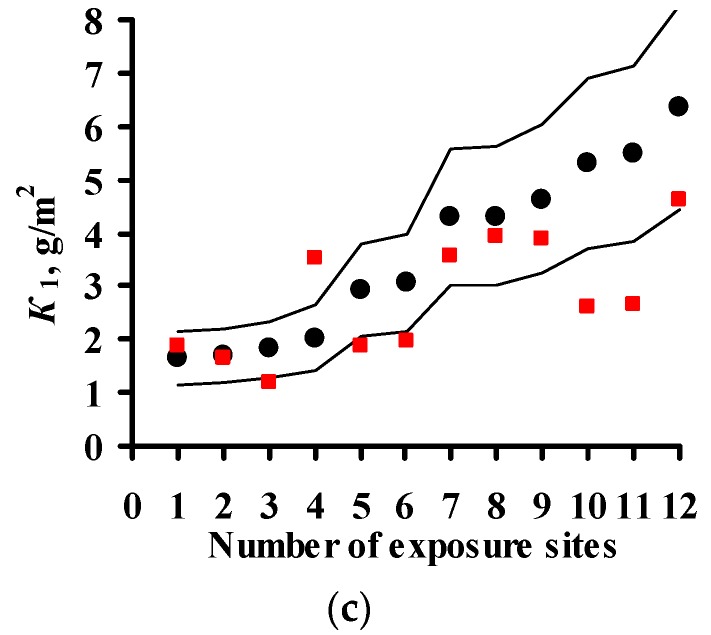
Zinc. RF program. *K*_1_ predictions by the New DRF (**a**); Standard DRF (**b**); and Unified DRF (**c**). ●—experimental *K*_1_ data; ■—*K*_1_ predictions. Thin lines show the calculation error (±30%). The numbers of the exposure sites are given in accordance with Table 4.

The results on *K*_1_^pr^ for zinc for the UN/ECE program, MICAT project, and RF program are presented in Figure 8, Figure 9 and Figure 10, respectively. In the UN/ECE program, the differences between the *K*_1_^pr^ and *K*_1_^exp^ values for zinc are more considerable than those for carbon steel. This may be due not only to the imperfection of the DRFs and the inaccuracy of the parameters and *K*_1_^exp^, but also to factors unaccounted for in DRFs that affect zinc. For all the DRFs, the *K*_1_^pr^ values match *K*_1_^exp^ to various extent; some of the latter exceed the error *δ* (±30%). Let us estimate the discrepancy between *K*_1_^pr^ and *K*_1_^exp^ for those *K*_1_^pr^ that exceed *δ*. For the New DRF (Figure 8a) and the Standard DRF (Figure 8b), overestimated *K*_1_^pr^ values are observed for low and medium *K*_1_^exp^, while underestimated ones are observed for medium and high *K*_1_^exp^. In general, the deviations of *K*_1_^pr^ from *K*_1_^exp^ are symmetrical for these DRFs, but the scatter of *K*_1_^pr^ is greater for the Standard DRF. For Unified DRF (Figure 8c), *K*_1_^pr^ are mostly overestimated, considering that the ∆*K*^[H+]^ = 0.029*Rain*[H^+^] component was not taken into account for some test locations due to the lack of data on [H^+^]. The ∆*K*^[H+]^ value can be significant, e.g., 2.35 g/m^2^ in US39 or 5.13 g/m^2^ in CS2.

With regard to the MICAT project, the New and Unified DRFs (Figure 9a,c) give overestimated *K*_1_^pr^ at low *K*_1_^exp^, but the Standard DRF gives *K*_1_^pr^ values comparable to *K*_1_^exp^ (Figure 9b). Starting from test locations No. 33–No. 36, the *K*_1_^pr^ values for all the DRFs are underestimated or significantly underestimated. It is evident from Figure 2b that rather many test locations with small [SO_2_] have extremely high *K*_1_^exp^. This fact confirms the uncertainty of experimental data from these locations, as shown for carbon steel as well. The following test locations can be attributed to this category: A3 (No. 43, No. 44, No. 53), B10 (No. 50), B11 (No. 49), B12 (No. 57), CO2 (No. 55, No. 58, No. 60), CO3 (No. 54, No. 61), PE6 (No. 36, No. 38), and M3 (No. 35, No. 59). There is little sense in making *K*_1_ predictions for these locations.

For the RF program, the *K*_1_^pr^ values calculated by the New and Unified DRFs are more comparable to *K*_1_^exp^ than those determined using the Standard DRF (Figure 10).

### 3.5. Analysis of DRFs for Zinc

As for steel, DRFs were analyzed by comparison of their coefficients. The nonlinear DRFs for zinc can be represented in the form:

*K*_1_ = *A* × [SO_2_]*^α^*× exp{*k*_1_ × *RH* + *k*_2_ × (*T*−10) + *k*_3_ × *Prec*}+ *B* × *Rain* × [H^+^]

or

*K*_1_ = *A* × [SO_2_]*^α^* × e*^k^*^1·*RH*^ × e*^k^*^2·(*T*−10)^ × e*^k^*^3·*Prec*^ + *B* × *Rain* × [H^+^].

The values of the coefficients used in Equations (4), (6) and (10) are presented in Table 8.

To compare the *α* values, *K*_1_ = 4 g/m^2^ at [SO_2_] = 1 μg/m^3^ was used for all DRFs. Let us note that the value *K*_1_ = 4 g/m^2^ was obtained during the estimation of *K* = *f*(SO_2_) for the development of the New DRF. The plots for all the programs are presented in Figure 2. For the New DRF, the line at *α* = 0.28 mostly passes through the average experimental points. For the Standard DRF, *α* = 0.44 is overestimated considerably, which may result in overestimated *K*_1_^pr^, especially at high [SO_2_]. For the Unified DRF at *α* = 0.22, the line passes, on average, slightly below the experimental points. The low *α* value, as for carbon steel, does not give a wide range of *K* values as a function of [SO_2_], which may result in underestimated *K*_1_^pr^, especially at high [SO_2_].

**Figure 11:**
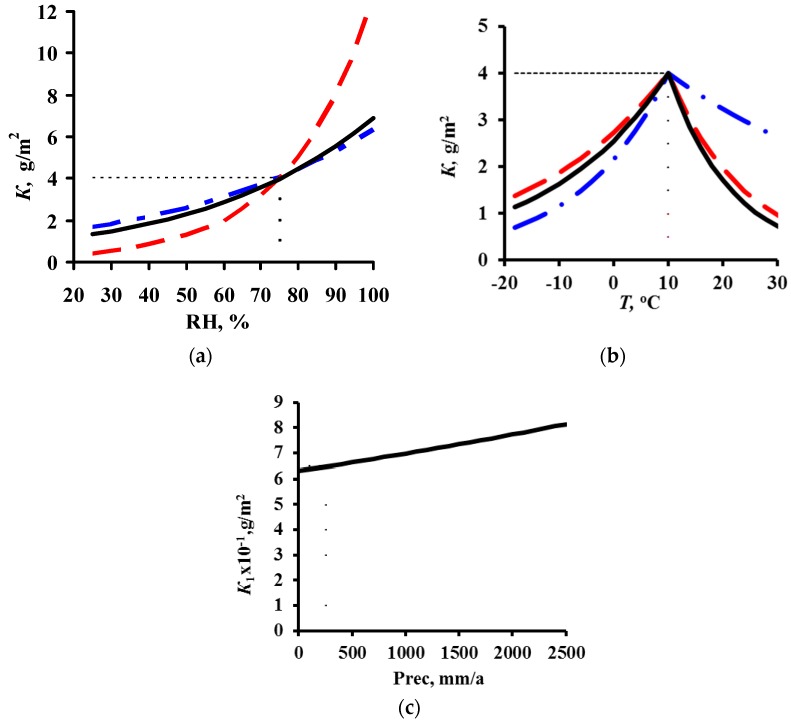
Variation of *K* for zinc versus relative humidity (**a**), temperature (**b**) and *Prec* (**c**) with account for the values of the DRF coefficients. ▬▬ by the New DRF; ▬ ▬ by the Standard DRF; ▬•▬ by the Unified DRF.

Let us assume for a comparative estimate of *k*_1_ and *k*_2_ that *K* = 4 g/m^2^ in a clean atmosphere at *T*_lim_ = 10 °C and *RH* = 75%. Figure 11 demonstrates the plots of *K* versus these parameters under these starting conditions. The Standard DRF (*k*_1_ = 0.46) shows an abrupt variation in *K* vs. *RH*. According to this relationship, at the same temperature, the *K* value should be 0.5 g/m^2^ at *RH* = 30% and 12.6 g/m^2^ at *RH* = 100%. According to the New DRF and Unified DRF with *k*_1_ = 0.22 and 0.18, respectively, the effect of *RH* is weaker, therefore *K* = 1.5 and 1.8 g/m^2^ at *RH* = 30%, respectively, and *K* = 6.9 and 6.4 g/m^2^ at *RH* = 100%, respectively.

The effect of temperature on *K* is shown in Figure 11b. In the New DRF, *k*_2_ = 0.045 at *T* ≤ 10 °C has an intermediate value; at *T* > 10 °C, *k*_2_ = −0.085 has the largest absolute value, which corresponds to an abrupt decrease in *K* with an increase in temperature. In the Unified DRF, *k*_2_ = −0.021 at *T* > 10 °C, i.e., an increase in temperature results in a slight decrease in *K*. As for the effect of *A*, this also contributes to higher *K*_1_^pr^ values despite the small *α* value.

In the Standard DRF, the value *A* = 0.0929 (g/m^2^), which is ~8 times smaller than in the New DRF, and a small *k*_2_ = −0.71 at *T* > 10 °C were taken to compensate the *K*_1_^pr^ overestimation due to the combination of high values, *α* = 0.44 and *k*_1_ = 0.46. In the Unified DRF, the high *A* value that is ~2 times higher than in the New DRF is not compensated by the combination of the low values, *α* = 0.22 and *k*_2_ = −0.021 at *T* > 10 °C. Therefore, the *K*_1_^pr^ values are mostly overestimated, Figure 8c and Figure 9c for trusted test locations. However, small *K*_1_^pr^ values were attained for low *T* at *k*_2_ = 0.62, Figure 10c.

The effect of *Prec* on *K* at *k*_3_ = 0.0001, which is taken into account only in the New DRF, given under the assumption that *K* = 0.65 in a clean atmosphere at *Prec* (*Rain*) = 250 mm/year, *T* = 15 °C and *RH* = 60% (e.g., location E5 in the MICAT project), is shown in Figure 11c. Upon an increase in *Prec* (*Rain*) from 250 to 2500 mm/year, *K* can increase from 0.65 to 0.81 g/m^2^.

As for carbon steel, the above analysis of coefficients in the DRFs for zinc confirms that the coefficients can be varied to obtain reliable *K*_1_^pr^ values. The New DRF based on *K* = *f*(SO_2_) gives the most reliable *K*_1_^pr^ values for zinc.

## 4. Estimation of Coefficients in DRFs for Carbon Steel and Zinc

Let us first note that the starting conditions that we took to demonstrate the effect of various atmosphere corrosivity parameters on *K* of carbon steel and zinc (Figure 7 and Figure 11) may not match the real values. However, the plots obtained give an idea on *K* variations depending on the coefficients in the DRFs.

For continental test locations under all programs, the *K*_1_^exp^ values are within the following ranges: for carbon steel, from 6.3 (Oimyakon, RF program) to 577 g/m^2^ (CS3, UN/ECE program); for zinc, from 0.65 (E5, MICAT project) to 16.41 g/m^2^ (CS3, UN/ECE program). That is, the difference in the corrosion losses is at least ~10–35 fold, the specific densities of these metals being nearly equal. Higher *K*_1_^pr^ values for steel than for zinc are attained using different coefficients at the parameters in the DRFs.

In the New DRFs, *A* is 7.7 and 0.71 g/m^2^ for carbon steel and zinc, respectively, i.e., the difference is ~10-fold. Higher *K*_1_^pr^ values for steel than for zinc were obtained chiefly due to the contribution of [SO_2_]*^α^* at *α* = 0.47 and 0.28, respectively. The values of *RH* and *Prec* affect the corrosion of steel more strongly than they affect zinc corrosion. The coefficients for these parameters are: *k*_1_ = 0.024 and 0.022; *k*_3_ = 0.00056 and 0.0001 for steel and zinc, respectively. However, the temperature coefficients (*k*_2_ = 0.095 and −0.095 for steel; *k*_2_ = 0.045 and −0.085 for zinc) indicate that, with a deviation of *T* from 10 °C, the corrosion process on steel is hindered to a greater extent than on zinc.

In the Standard DRF, *A* is 1.77 and 0.0129 μm for carbon steel and zinc, respectively, i.e., the difference is ~137-fold. The *α* value for steel is somewhat higher than that for zinc, i.e., 0.52 and 0.44 respectively, which increases the difference of *K*_1_^pr^ for steel from that for zinc. As shown above, the difference should not be greater than 35-fold. This difference is compensated by the 2.3-fold higher effect of *RH* on zinc corrosion than on steel corrosion (*k*_1_ = 0.046 and 0.020 for zinc and steel, respectively). Furthermore, the temperature coefficient *k*_2_ at *T* ≤ 10 °C for steel is 3.95 times higher than that for zinc. This indicates that steel corrosion slows down abruptly in comparison with zinc as *T* decreases below 10 °C. At *T* > 10 °C, the *k*_2_ values for steel and zinc are comparable. Taking the values of the coefficients presented into account, the *K*_1_^pr^ values for steel are 15-fold higher, on average, than those for zinc at *T* ≤ 10 °C, but ~60-fold at *T* > 10 °C. Of course, this is an approximate estimate of the coefficients used in the Standard DRF.

In the Unified DRF, *A* is 3.54 and 0.188 μm for carbon steel and zinc, respectively, i.e., the difference is ~19-fold. The *α* value for steel is lower than that for zinc, i.e., 0.13 and 0.22 respectively, which decreases the difference of *K*_1_^pr^ of steel from that of zinc. Conversely, the *RH* value affects steel corrosion somewhat more strongly than that of zinc (*k*_1_ = 0.020 and 0.018 for steel and zinc, respectively). The *k*_2_ values for steel and zinc are comparable in both temperature ranges. The ∆*K*^[H+]^ component was introduced only for zinc, which somewhat complicates the comparison of the coefficients in these DRFs.

All the presented DRFs are imperfect not only because of the possible inaccuracy of the mathematical expressions as such, but also due to the inaccuracy of the coefficients used in the DRFs. The *K*_1_^pr^ values obtained using the New DRF match *K*_1_^exp^ most accurately. However, while the *α* values that were assumed to be 0.47 and 0.28 for carbon steel and zinc, respectively, may be considered as accurate in a first approximation, the other coefficients need to be determined more accurately by studying the effect of each atmosphere corrosivity parameter on corrosion, with the other parameters being unchanged. Studies of this kind would allow each coefficient to be estimated and DRFs for reliable prediction of *K*_1_ in atmospheres with various corrosivity to be created.

## 5. Conclusions

1. *K* = *f*(SO_2_) plots of corrosion losses of carbon steel and zinc vs. sulfur dioxide concentration were obtained to match, to a first approximation, the mean meteorological parameters of atmosphere corrosivity.
2. Based on the *K* = *f*(SO_2_) relationships obtained, with consideration for the nonlinear effect of temperature on corrosion, New DRFs for carbon steel and zinc in continental territories were developed.
3. Based on the corrosivity parameters at test locations under the UN /ECE and RF programs and the MICAT project, predictions of first-year corrosion losses of carbon steel and zinc were given using the New DRF, Standard DRF, and Unified DRF, as well as the linear model for carbon steel obtained in [20] with the aid of an artificial neural network. The predicted corrosion losses are compared with the experimental data for each DRF. It was shown that the predictions provided by the New DRFs for the first-year match the experimental data most accurately.
4. An analysis of the values of the coefficients used in the DRFs for the prediction of corrosion losses of carbon steel and zinc is presented. It is shown that more accurate DRFs can be developed based on quantitative estimations of the effects of each atmosphere corrosivity parameter on corrosion.

## Figures and Tables

**Table 1:** Countries, names, and codes of test locations.

MICAT Project			UN/ECE Program		
Country	Test Location	Designation	Country	Test Location	Designation
Argentina	Villa Martelli	A2	Czech Republic	Prague	CS1
Argentina	Iguazu	A3	Czech Republic	Kasperske Hory	CS2
Argentina	San Juan	A4	Czech Republic	Kopisty	CS3
Argentina	La Plata	A6	Finland	Espoo	FIN4
Brasil	Caratinga	B1	Finland	Ähtäri	FIN5
Brasil	Sao Paulo	B6	Finland	Helsinki Vallila	FIN6
Brasil	Belem	B8	Germany	Waldhof Langenbrügge	GER7
Brasil	Brasilia	B10	Germany	Aschaffenburg	GER8
Brasil	Paulo Afonso	B11	Germany	Langenfeld Reusrath	GER9
Brasil	Porto	B12	Germany	Bottrop	GER10
Colombia	San Pedro	CO2	Germany	Essen Leithe	GER11
Colombia	Cotove	CO3	Germany	Garmisch Partenkirchen	GER12
Ecuador	Guayaquil	EC1	Netherlands	Eibergen	NL18
Ecuador	Riobamba	EC2	Netherlands	Vredepeel	NL19
Spain	Leon	E1	Netherlands	Wijnandsrade	NL20
Spain	Tortosa	E4	Norway	Oslo	NOR21
Spain	Granada	E5	Norway	Birkenes	NOR23
Spain	Arties	E8	Sweden	Stockholm South	SWE24
Mexico	Mexico (a)	M1	Sweden	Stockholm Centre	SWE25
Mexico	Mexico (b)	M2	Sweden	Aspvreten	SWE26
Mexico	Cuernavaca	M3	Spain	Madrid	SPA31
Mexico	San Luis Potosi	PE4	Spain	Toledo	SPA33
Peru	Arequipa	PE5	Russian Federation	Moscow	RUS34
Peru	Arequipa	PE6	Estonia	Lahemaa	EST35
Peru	Pucallpa	U1	Canada	Dorset	CAN37
Uruguay	Trinidad	U3	USA	Research Triangle Park	US38
-	-	-	USA	Steubenville	US39

**Table 2:** Atmosphere corrosivity parameters of test locations, first-year corrosion losses of carbon steel and zinc (*K*_1_, g/m^2^) under the UN/ECE program, and numbers of test locations in the order of increasing *K*_1_.

Designation	*T*, °C	*RH*, %	*TOW*, Hours/a	*Prec*, mm/a	[SO_2_], μg/m^3^	[H^+^], mg/L	Steel		Zinc	
							g/m^2^	No.	g/m^2^	No.
CS1	9.5	79	2830	639.3	77.5	-	438.0	76	14.89	92
CS1	10.3	74	2555	380.8	58.1	0.0221	-	-	6.98	45
CS1	9.1	73	2627	684.3	41.2	0.0714	270.7	64	7.78	53
CS1	9.8	77	3529	581.1	32.1	0.0342	241.0	58	5.69	31
CS2	7.0	77	3011	850.2	19.7	-	224.0	51	8.95	65
CS2	7.4	76	3405	703.4	25.6	0.045	-	-	7.99	58
CS2	6.6	73	2981	921	17.9	0.1921	152.9	33	6.77	44
CS2	7.2	74	3063	941.2	12.2	0.0366	148.2	30	3.46	4
CS3	9.6	73	2480	426.4	83.3	-	557.0	77	16.41	94
CS3	9.9	72	2056	416.6	78.4	0.0242	-	-	11.59	87
CS3	8.9	71	2866	431.6	49	0.058	350.2	73	11.74	88
CS3	9.7	75	2759	512.7	49.2	0.0567	351.8	74	12.17	89
FIN4	5.9	76	3322	625.9	18.6	-	271.0	63	-	-
FIN4	6.4	80	4127	657	13.9	0.0392	-	-	8.42	62
FIN4	5.6	79	3446	754.6	2.3	0.0231	130.3	21	5.18	25
FIN4	6.0	80	3607	698.1	2.6	0.0334	120.9	20	4.68	19
FIN5	3.1	78	2810	801.3	6.3	-	132.0	23	8.92	66
FIN5	3.9	80	3342	670.7	1.8	0.0271	-	-	7.70	52
FIN5	3.4	81	2994	609.7	0.9	0.0201	48.4	4	6.62	41
FIN5	3.9	83	3324	675.4	0.8	0.0247	59.3	5	4.61	16
FIN6	6.3	78	3453	673.1	20.7	-	273.0	65	-	-
FIN6	6.8	80	4017	665.6	15.3	0.0554	-	-	9.29	70
FIN6	6.2	78	3360	702.4	4.8	0.0221	162.2	34	5.69	33
FIN6	6.6	76	3288	649.2	5.5	0.0139	195.8	44	5.62	30
GER7	9.3	80	4561	630.6	13.7	-	264.0	62	-	-
GER7	10.2	80	4390	499.7	11	0.0358	-	-	7.85	56
GER7	8.9	81	4382	624.4	8.2	0.0342	230.9	53	9.07	68
GER7	9.5	81	4676	595.6	3.9	0.0265	166.1	36	4.25	13
GER8	12.3	77	4282	626.9	23.7	-	213.0	48	-	-
GER8	12.2	67	2541	655.4	14.2	0.0411	-	-	4.68	18
GER8	11.4	64	3563	561.2	12.6	0.0183	116.2	17	5.18	26
GER8	11.6	65	2359	779	9.6	-	141.2	27	4.10	12
GER9	10.8	77	4220	782.9	24.5	-	293.0	69	-	-
GER9	11.7	80	4940	697.6	20.3	0.0366	-	-	6.62	40
GER9	10.7	79	4437	619.1	16.3	0.0291	230.9	54	9.07	69
GER9	11.4	81	5210	841	11.1	0.0278	209.8	47	7.63	-
GER10	11.2	75	4077	873.8	50.6	-	373.0	75	-	-
GER10	12	76	4107	696.6	48.5	0.0253	-	-	10.66	81
GER10	10.3	78	4201	707.3	41.6	0.0211	347.1	72	15.34	93
GER10	11.8	80	4930	912.9	30.2	0.0334	294.1	70	7.85	55
GER11	10.5	79	4537	713.1	30.3	-	342.0	71	-	-
GER11	11.5	77	4040	644.5	25.6	0.042	-	-	9.72	73
GER11	10.1	79	4120	683.6	22.9	0.0253	293.3	68	11.45	86
GER11	10.9	78	4632	889.3	16.2	0.0247	241.0	57	7.06	46
GER12	8.0	82	4989	1491.5	9.4	-	133.0	24	8.35	61
GER12	7.3	82	4201	1183.1	6.1	0.0171	-	-	7.27	49
GER12	7.1	84	4545	1552.4	3.2	0.0018	89.7	9	7.20	48
GER12	7.4	83	4375	1503	2.4	-	85.0	8	3.74	9
NL18	9.9	83	5459	904.2	10.1	-	232.0	55	9.93	76
NL18	10.9	79	4482	705.9	8.5	0.0046	-	-	8.14	59
NL18	9.5	82	4808	872.8	7.4	0.004	204.4	45	7.92	57
NL18	10.3	83	5358	987.1	4.7	0.0366	144.3	28	4.75	20
NL19	10.3	81	5354	845	13	-	283.0	66	-	-
NL19	11	81	4969	569.1	9.9	0.0049	-	-	9.07	67
NL19	10	82	5084	749.2	8.3	0.0021	238.7	56	11.09	84
NL19	10.9	83	5454	828.9	4.5	-	180.2	39	-	-
NL20	10.3	81	5125	801.3	13.7	-	259.0	59	-	-
NL20	11.1	77	4424	608.8	10.3	0.0106	-	-	10.22	77
NL20	10.1	81	4688	679.6	9.3	0.0113	205.1	46	11.38	85
NL20	11.1	82	5141	789.9	5.8	0.0038	172.4	37	6.34	37
NOR21	7.6	70	2673	1023.8	14.4	-	229.0	52	-	-
NOR21	8.8	70	2864	526.6	7.9	0.0326	-	-	5.69	32
NOR21	7.7	68	2471	440.1	6	0.0156	134.9	25	6.70	43
NOR21	7.5	69	2827	680	2.9	0.0136	100.6	11	3.53	7
NOR23	6.5	80	4831	2144.3	1.3	-	194.0	43	-	-
NOR23	7.4	77	4193	1762.2	0.9	0.042	-	-	8.50	63
NOR23	5.9	75	3341	1188.6	0.7	0.0374	131.8	22	10.58	80
NOR23	6.4	76	3779	1419.7	0.7	0.0326	109.2	15	5.04	24
SWE24	7.6	78	3959	531	16.8	-	264.0	61	10.36	79
SWE24	8.7	70	3074	473.2	8.4	0.0366	-	-	6.12	35
SWE24	7	70	2580	577	5.7	0.043	120.1	18	4.54	15
SWE24	7.5	73	3160	580.6	4.2	0.0231	103.0	13	4.25	14
SWE25	7.6	78	3959	531	19.6	-	263.0	60	9.76	74
SWE25	8.7	70	3074	473.2	10.3	0.0366	-	-	5.62	29
SWE25	7	70	2580	577	4.7	0.043	103.0	12	3.53	5
SWE25	7.5	73	3160	580.6	3.4	0.0231	95.2	10	3.53	8
SWE26	6.0	83	4534	542.7	3.3	-	147.0	29	8.31	60
SWE26	7.6	77	3469	342.3	2	0.043	-	-	6.70	42
SWE26	6	81	3592	467.8	1.3	0.043	74.9	6	4.90	23
SWE26	6.8	82	4118	525.2	1.1	0.0278	81.1	7	6.05	34
SPA31	14.1	66	2762	398	18.4	-	222.0	50	7.74	54
SPA31	15.2	56	1160	331.5	15.3	0.0073	-	-	4.82	22
SPA31	14.3	67	2319	360.1	8.2	0.0003	162.2	35	3.53	6
SPA31	15.7	68	2766	223.9	7.8	0.0002	151.3	32	2.30	2
SPA33	14.0	64	2275	785	3.3	-	45.0	3	3.37	3
SPA33	15.5	61	2147	610.4	13.5	0.0006	-	-	3.89	11
SPA33	13.4	61	1888	432.5	1.7	0.0012	25.7	1	3.89	10
SPA33	14.8	57	1465	327.4	4.2	0.0006	35.9	2	1.66	1
RUS34	5.5	73	2084	575.4	19.2	-	181.0	40	10.32	78
RUS34	5.7	76	2894	860.2	30.8	0.0006	-	-	8.64	64
RUS34	5.7	74	2444	880.6	28.7	0.0009	141.2	26	6.48	39
RUS34	5.6	71	1514	666.7	16.4	0.0008	120.9	19	4.61	17
EST35	5.5	83	4092	447.8	0.9	-	185.0	41	7.18	47
EST35	6.7	81	4332	532.7	0.6	0.0226	-	-	9.43	71
CAN37	5.5	75	3252	961.1	3.3	-	149.0	31	9.88	75
CAN37	5	79	3431	1103	3	0.042	-	-	6.26	38
CAN37	4.3	80	3302	1080	2.1	0.0482	110.0	16	5.26	27
CAN37	5.2	80	3386	1022.8	3.3	0.0461	103.7	14	6.19	36
US38	14.6	69	3178	846.7	9.6	-	176.0	38	10.72	82
US38	16.3	66	3026	1106.7	9.2	0.0358	-	-	12.46	90
US38	15.5	64	2644	982.3	10.1	0.0349	184.9	42	9.72	72
US38	15.8	68	-	1037.6	9.3	0.0482	-	-	4.75	21
US39	12.3	67	2111	733.1	58.1	-	214.0	49	13.61	91
US39	11.2	61	1391	967.4	55.2	0.0838	-	-	11.02	83
US39	11.8	65	1532	729.4	43.1	0.0941	290.2	67	7.34	50
US39	11.8	69	-	756.8	38.3	0.0765	-	-	5.26	28

**Table 3:** Atmosphere corrosivity parameters of test locations, first-year corrosion losses of carbon steel and zinc (*K*_1_, g/m^2^) under the MICAT program and those reported in [20], and numbers of test locations in the order of increasing *K*_1_. Adapted from [20], with permission from © 2000 Elsevier.

Designation	*T*, °C	*RH*, %	*Rain*, mm/a	[SO_2_], μg/m^3^	Cl^−^, mg/(m^2^·Day)	*TOW*, h/a	Steel		Zinc	
							g/m^2^	No.	g/m^2^	No.
A2 *	16.7	75	1729	10	Ins	5063	122.5	36 (34)	8.06	41
A2	17.1	72	983	10	Ins	4222	125.6	38	7.56	39
A2	17.0	74	1420	9	Ins	4862	96.7	25	10.15	47
A3	20.6	76	2158	Ins (5) **	Ins (1.5)	5825	44.5	12 (11)	14.76	53
A3	20.9	74	2624	Ins (5)	Ins (1.5)	5528	45.2	13 (12)	8.42	43
A3	22.1	75	1720	Ins (5)	Ins (1.5)	5545	43.7	10 (9)	8.50	44
A4	18.0	51	35	Ins (5)	Ins (1.5)	999	35.9	6 (6)	2.02	15
A4	20.0	49	111	Ins (5)	Ins (1.5)	850	35.1	5 (5)	0.94	3
A4	18.3	51	93	Ins (5)	Ins (1.5)	867	43.7	11 (10)	1.58	10
A6	17.0	78	1178	6.22	Ins	5195	197.3	55 (51)	5.54	28
A6 *	16.7	77	1263	8.21	Ins	4949	224.6	59 (55)	6.70	32
A6 *	16.6	78	1361	6.2	Ins	5528	234.8	61 (57)	7.49	37
B1	21.2	75	996	1.67	1.57	4222	102.2	28(26)	4.32	26
B6	19.7	75	1409	67.2 (28)	Ins (1.5)	5676	113.9	31 (29)	8.57	45
B6	19.5	76	1810 (1910)	66.8 (28)	Ins (1.5)	5676	182.5	53 (49)	10.66	48
B6	19.6	75	1034	48.8 (28)	Ins (1.5)	5676	188.8	54 (50)	6.98	34
B8	26.1	88	2395	Ins (5)	Ins (1.5)	5974	151.3	44 (40)	7.92	40
B10	20.4	69 (72)	1440	Ins (5)	Ins (1.5)	3872	100.6	26 (24)	12.82	50
B11	25.9	77	1392	Ins	Ins	1507	134.9	41	11.52	49
B12	26.6	90	2096	Ins	Ins	4222	38.2	8	23.83	57
CO2	9.6 (14.1)	98 (81)	1800	0.56 (5)	Ins (1.5)	8760 (7008)	106.9	30 (28)	24.48	58
CO2	11.4	90	1800	0.56 (5)	Ins (1.5)	8760 (7808)	138.1	42 (38)	25.78	60
CO2	13.5 (14.2)	81 (73)	1800	0.56 (5)	Ins (1.5)	8760 (7808)	152.9	46 (42)	20.88	55
CO3 *	27.0	76	900	0.33	Ins	2891	120.9	35 (33)	18.65	54
CO3 *	27.0	76	900	0.33	Ins	2891	204.4	57 (53)	27.00	61
CO3 *	27.0	76	900	0.33	Ins	2891	132.6	40 (37)	25.56	59
EC1	26.1	71	936	4.20	1.5	4853	152.1	45 (41)	1.08	5
EC1	26.9	82	635	2.72	1.31	5790	176.3	52 (48)	1.15	6
EC1 *	24.8	75	564	2.1	1.66	3101	201.2	56 (52)	2.38	17
EC2	12.9	66	554	1.0	0.4	3583	60.8	17 (16)	-	-
EC2 *	13.2	71	598	1.35	1.14	4932	70.2	21 (20)	-	-
E1	12.0	69	652	1.18 (16.2)	1.5	3364	158.3 (150.5)	48 (44)	3.02	20
E1 *	10.6	65	495	1.18	1.5	2374	175.5	51 (47)	2.88	18
E1	11.1	63	334	1.18 (16.2)	1.5	2111	153.7	47 (43)	2.09	16
E4	18.1	65	554	8.3	1.5	3416	158.3	49 (45)	1.94	14
E4	17.0	63	521	5.7	1.5	2646	151.3	43 (39)	1.51	8
E4	17.2	62	374	1.9	1.5	2768	163.8	50 (46)	1.94	13
E5	16.3	59	416	10.3	1.5	1323	95.9	24 (23)	1.01	4
E5	15.0 (15.8)	59 (58)	258 (239)	5.4	1.5	1104	53.0	16 (15)	0.65	2
E5	15.6	58	266	2.8	1.5	2400	49.9	15 (14)	0.65	1
E8	8.8	52 (72)	738	9.1	1.8	876	25.7	3 (3)	1.66	11
E8	6.9	52 (72)	624	8.9	1.6	876	28.1	4 (4)	1.22	7
E8	7.8	52 (72)	681	9.0	1.7	876	37.4	7 (7)	3.10	21
M1	16.0	62	743	15.6	1.5	2523 (2321)	120.1	34 (32)	5.83	29
M1	14.8 (15.2)	66 (65)	747	7.7 (5.6)	1.5	2523	67.1	20 (19)	5.98	31
M1	15.4	64 (63)	747	17.5	1.5	2523 (2427)	39.8	9 (8)	5.83	30
M2	21.0	56	1352	6.7	1.5	1664	118.6	33 (31)	8.35	42
M2	21.0	56	1724	9.9	Ins (1.5)	1857	88.9	22 (21)	14.33	52
M2	21.0	56	1372	7.1	Ins (1.5)	1752	106.9	29 (27)	6.84	33
M3	18.0	51	374	31.1	Ins	1410	292.5	62 (58)	10.01	46
M3 *	18.0	62	374	10.9	Ins	1410	205.9	58 (54)	21.24	56
M3 *	18.0	60	374	14.6	Ins	2646	229.3	60 (56)	7.06	35
PE4	16.4	37	17	Ins (5)	Ins (1.5)	26	117.0	32 (30)	1.66	12
PE4	17.2	33	34 (89)	Ins (5)	Ins (1.5)	175 (26)	128.7	39 (36)	1.58	9
PE5	12.2	67	632	Ins (0)	Ins (0)	2847	7.8	1 (1)	3.89	23
PE5	12.2	67	672 (792)	Ins (0)	Ins (0)	2689 (2847)	13.3	2 (2)	2.88	19
PE6	25.4	84	1523	Ins (5)	Ins (1.5)	5037 (4580)	122.5	37 (35)	7.06	36
PE6	25.8	83	1158 (1656)	Ins (5)	Ins (1.5)	5790 (4380)	100.6	27 (25)	7.49	38
U1	16.8	74	1182	0.6 (1)	1.8 (2.2)	5133	64.0	19 (18)	4.03	24
U1 *	16.6	73	1324	0.8	1.2	4976	62.4	18 (17)	3.74	22
U1 *	16.7	76	1306	Ins	Ins	4792	47.6	14 (13)	4.10	25
U3 *	17.7	79	1490	Ins	Ins	5764	94.4	23 (22)	4.39	27
CH1	14.2	71	355	20	2.18	3469	221.5	63	12.89	51

* the test locations not used in [20]; ** the values reported in [20] are shown in parentheses.

**Table 4:** Atmosphere corrosivity parameters of test locations and first-year corrosion losses of carbon steel and zinc (*K*_1_, g/m^2^) in Russian Federation test locations and their numbers in the order of increasing *K*_1_.

Test Location	*T*, °C	*RH*, %	*Prec*, mm/a	[SO_2_], μg/m^3^	Steel		Zinc	
					g/m^2^	No.	g/m^2^	No.
Bilibino	−12.2	80	218	3	5.4	1	1.64	1
Oimyakon	−16.6	71	175	3	8.1	2	1.81	3
Ust-Omchug	−11	70	317	5	12.4	3	2.91	5
Atka	−12	72	376	3	15.2	4	1.69	2
Susuman	−13.2	71	283	10	17.0	5	3.07	6
Tynda	−6.5	72	525	5	21.2	6	5.30	10
Klyuchi	1.4	69	253	3	23.4	7	2.03	4
Aldan	−6.2	72	546	5	24.6	8	5.47	11
Pobedino	−0.9	77	604	3	36.5	9	4.30	7
Yakovlevka	2.5	70	626	3	40.6	10	4.64	9
Pogranichnyi	3.6	67	595	3	49.0	11	4.32	8
Komsomolsk-on-Amur	−0.7	76	499	10	63.2	12	6.35	12

**Table 5:** Atmosphere corrosivity parameters and first-year corrosion losses of carbon steel in test locations. Adapted from [19], with permission from © 1999 Elsevier.

[SO_2_], μg/m^3^	Cl^−^, mg/(m^2^·Day)	*K*_1_, g/m^2^
3	2	137.7
5	0,3	46.1
5	0,7	130.7
8	1	137.7
8	0	140.0
14	2	193.8
15	2	228.4
15	1	236.1
17	0,16	136.1
26	1	236.1
32	2	276.1
116	0,62	232.2

**Table 6:** Atmosphere corrosivity parameters and first year corrosion losses of carbon steel in certain test locations under the MICAT project.

Locations with Uncertain Data									Locations with Trusted Data								
Designation	No.	*T*, °C	*RH*, %	*TOW*, 1/a	*Prec*, mm/a	[SO_2_], μg/m^3^	*K*_1_^exp^		Designation	No.	*T*, °C	*RH*, %	*TOW*, 1/a	*Prec*, mm/a	[SO_2_], μg/m^3^	*K*_1_^exp^	
							µm	g/m^2^								µm	g/m^2^
PE4	32	16.4	37	0.003	17	1	15.0	117.0	E8	3	8.8	52	0.100	738	9.1	3.3	25.7
PE4	39	17.2	33	0.020	34	1	16.5	128.7	E8	4	6.9	52	0.100	624	8.9	3.6	28.1
A4	5	20.0	49	0.097	111	1	4.5	35.1	E8	7	7.8	52	0.100	681	9	4.8	37.4
A4	6	18.0	51	0.114	35	1	4.6	35.9	M2	29	21.0	56	0.200	1372	7.1	13.7	106.9
M3	58	18.0	62	0.161	374	10.9	26.4	205.9	M2	33	21.0	56	0.190	1352	6.7	15.2	118.6
M3	62	18.0	51	0.161	374	31.1	37.5	292.5	M2	22	21.0	56	0.212	1724	9.9	11.4	88.9
M3	60	18.0	60	0.302	374	14.6	29.4	229.3	E5	15	15.6	58	0.161	266	2.8	6.4	49.9
E1	47	11.1	63	0.241	334	1.18	19.7	153.7	E5	16	15.0	59	0.126	258	5.4	6.8	53.0
E1	48	12.0	69	0.384	652	1.18	20.3	158.3	M1	34	16.0	62	0.288	743	15.6	15.4	120.1
E1	51	10.6	65	0.271	495	1.18	22.5	175.5	M1	9	15.4	64	0.288	743	17.5	5.1	39.8
E4	43	17.0	63	0.302	521	5.7	19.4	151.3	M1	20	14.8	66	0.288	743	7.7	8.6	67.1
E4	49	18.1	65	0.390	554	8.3	20.3	158.3	A2	38	17.1	72	0.482	983	10.0	16.1	125.6
E4	50	17.2	62	0.316	374	1.9	21.0	163.8	A2	36	16.7	75	0.578	1729	10.0	15.7	122.5
B10	26	20.4	69	0.442	1440	1	12.9	100.6	A2	25	17.0	74	0.555	1420	9	12.4	96.7
B1	28	21.2	75	0.484	996	1.67	13.1	102.2	A3	12	20.6	76	0.665	2158	1	5.7	44.5
CO3	40	27.0	76	0.330	900	1	17.0	132.6	A3	13	20.9	74	0.631	2624	1	5.8	45.2
CO3	57	27.0	76	0.330	900	1	26.2	204.4	A3	10	22.1	75	0.633	1720	1	5.6	43.7
B11	41	25.9	77	0.172	1392	1	17.3	134.9	-	-	-	-	-	-	-	-	-
EC1	56	24.8	75	0.354	564	2.1	25.8	201.2	-	-	-	-	-	-	-	-	-
EC1	52	26.9	82	0.661	635	2.72	22.6	176.3	-	-	-	-	-	-	-	-	-

**Table 7:** Values of coefficients used in the nonlinear DRFs for carbon steel.

DRF	*A*		*α*	*k*_1_	*k*_2_		*k*_3_
	µm	g/m^2^			*T* ≤ 10	*T* > 10	
New	0.99	7.7	0.47	0.024	0.095	−0.095	0.00056
Standard	1.77	13.8	0.52	0.020	0.150	−0.054	-
Unified	3.54	27.6	0.13	0.020	0.059	−0.036	-

**Table 8:** Values of coefficients used in the nonlinear DRFs for zinc.

DRF	*A*		*α*	*k*_1_	*k*_2_		*k*_3_	*B*	
	µm	g/m^2^			*T* ≤ 10	*T* > 10		µg	g/m^2^
New	0.0986	0.71	0.28	0.022	0.045	−0.085	0.0001	-	-
Standard	0.0129	0.0929	0.44	0.046	0.038	−0.071	-	-	-
Unified	0.188	1.35	0.22	0.018	0.062	−0.021	-	0.00403	0.029
